# Impaired oxidative stress response characterizes HUWE1-promoted X-linked intellectual disability

**DOI:** 10.1038/s41598-017-15380-y

**Published:** 2017-11-08

**Authors:** Matthias Bosshard, Rossana Aprigliano, Cristina Gattiker, Vuk Palibrk, Enni Markkanen, Paul Hoff Backe, Stefania Pellegrino, F. Lucy Raymond, Guy Froyen, Matthias Altmeyer, Magnar Bjørås, Grigory L. Dianov, Barbara van Loon

**Affiliations:** 1 Department of Molecular Mechanisms of Disease, University of Zurich, Zürich, 8057 Switzerland; 2 Department of Clinical and Molecular Medicine, Norwegian University of Science and Technology (NTNU), Trondheim, 7491 Norway; 3 Institute of Clinical Medicine, Faculty of Medicine, University of Oslo, Oslo, 0318 Norway; 4 Department of Medical Genetics, Cambridge Institute for Medical Research, Cambridge, CB2 0XY United Kingdom; 5 Human Genome Laboratory, Department of Human Genetics, Leuven, 3000KU Belgium; 6 Department of Pathology and Medical Genetics, St. Olavs Hospital, Trondheim University Hospital, Trondheim, 7491 Norway; 7 CRUK/MRC Institute for Radiation Oncology, Department of Oncology, University of Oxford, Oxford, OX3 7DQ United Kingdom; 8 Institute of Cytology and Genetics, Siberian Branch of the Russian Academy of Sciences, Novosibirsk, 630090 Russia

## Abstract

Mutations in the HECT, UBA and WWE domain-containing 1 (*HUWE1*) E3 ubiquitin ligase cause neurodevelopmental disorder X-linked intellectual disability (XLID). HUWE1 regulates essential processes such as genome integrity maintenance. Alterations in the genome integrity and accumulation of mutations have been tightly associated with the onset of neurodevelopmental disorders. Though *HUWE1* mutations are clearly implicated in XLID and HUWE1 regulatory functions well explored, currently much is unknown about the molecular basis of HUWE1-promoted XLID. Here we showed that the HUWE1 expression is altered and mutation frequency increased in three different XLID individual (HUWE1 p.R2981H, p.R4187C and *HUWE1* duplication) cell lines. The effect was most prominent in HUWE1 p.R4187C XLID cells and was accompanied with decreased DNA repair capacity and hypersensitivity to oxidative stress. Analysis of HUWE1 substrates revealed XLID-specific down-regulation of oxidative stress response DNA polymerase (Pol) λ caused by hyperactive HUWE1 p.R4187C. The subsequent restoration of Polλ levels counteracted the oxidative hypersensitivity. The observed alterations in the genome integrity maintenance may be particularly relevant in the cortical progenitor zones of human brain, as suggested by HUWE1 immunofluorescence analysis of cerebral organoids. These results provide evidence that impairments of the fundamental cellular processes, like genome integrity maintenance, characterize HUWE1-promoted XLID.

## Introduction

Intellectual disability (ID) is a complex neurodevelopmental condition that results in arrested or incomplete development of the mind^1,2^. It manifests before the age of 18 and has an estimated 2% prevalence in the Western world population. X-linked ID (XLID) is a subset of ID’s associated with alterations in over 100 different genes located on the X chromosome. To date more than twenty XLID families harboring either a submicroscopic duplication or specific missense mutations in the HECT, UBA and WWE domain-containing 1 (*HUWE1*) gene have been identified^3–7^. HUWE1-promoted XLID is characterized by high clinical heterogeneity, ranging from dysmorphic facial features and mild ID, to microcephaly, extreme ID, deafness, joint contractures and incontinence^3^.

*HUWE1* encodes for an 482-kDa E3 ubiquitin ligase^8^. Through poly-ubiquitination HUWE1 regulates numerous substrates, predominantly predisposing them for proteasomal degradation. In addition to its substrates, HUWE1 also mediates selfubiquitination; this is counteracted by Ser18-containing isoform of ubiquitin-specific-processing protease 7 (USP7S) that de-ubiquitinates HUWE1 and prevents its subsequent degradation^9–11^. By poly-ubiquitinating p53 and the important DNA damage response factors, like Cdc6 and DNA polymerases (Pols) λ and β, HUWE1 contributes to the maintenance of genome integrity^8,12,13^. Loss of HUWE1 was recently shown to cause increased genome instability^14^. Interestingly, growing number of evidence indicates that the genome integrity maintenance mechanisms are of key importance for the neuronal development^15^ and mutations in DNA repair genes have been identified as causative in IDs^16,17^.

*Huwe1* is critical developmental gene and its total loss leads to embryonic lethality in mice^18^. Conditional *Huwe1* inactivation in the mouse brain results in disorganization of laminar cortex patterning causing neonathal lethality^18,19^. Studies with the conditional *Huwe1* mice further underline the specific importance of HUWE1 for neuronal development, in particular for the initiation of cell cycle exit and start of neuronal differentiation in the brain^20^. In the mouse adult hippocampus HUWE1 is required for proliferating neural stem cells (NSCs) to return to quiescence^21^. Many of human XLID-causing HUWE1 mutations reside within, or are in the close vicinity of the catalytic HECT domain (p.R2981H, p.R4013W, p.R4187C and p.G4310R)^3,5^. Very recent work in *C*. *elegans* suggested that mutations corresponding to p.R2981H and p.R4187C potentially alter HUWE1 function^22^. Further, we showed that the levels of mutated HUWE1 p.G4310R are significantly lower in XLID individual cells when compared to cells from healthy individual^3^. Currently it remains however unknown, if other XLID-associated mutations result in similarly changed HUWE1 levels, as well as what is the impact of potentially altered expression and function on the HUWE1-regulated processes, such as genome integrity maintenance. Taken together, tough HUWE1 mutations are clearly implicated in XLID and HUWE1 regulatory functions well explored, much is unknown about the cellular processes that are altered by XLID-causing HUWE1 and that contribute to the onset of the disease.

In this study we addressed how mutated HUWE1 contributes to XLID development. Our results revealed increased mutation frequency in three different HUWE1-promoted XLID individual cell lines (with HUWE1 p.R2981H or p.R4187C, and *HUWE1* duplication). Mutation frequency was highest in the HUWE1 p.R4187C XLID cells and was accompanied with a decreased DNA repair capacity and hypersensitivity to oxidative stress. While accumulation of *de novo* mutations was previously shown to negatively affect functioning of developmental genes^23^, increased oxidative stress was suggested to have particular pathophysiological role in neurodevelopmental disorders, including XLID^24,25^. Immunoblot analysis of HUWE1 targets showed an XLID-specific down-regulation of oxidative stress response enzyme Polλ that was caused by the hyperactive HUWE1 p.R4187C. Further, use of a small molecule USP7 inhibitor in XLID cells and consequent restoration of Polλ levels counteracted the oxidative hypersensitivity. Analysis of cerebral organoids indicated a specific localization of human HUWE1 in structures resembling human cortical progenitor regions, thus supporting the role of HUWE1 in neurodevelopment. Based on these findings we propose that the XLID-specific HUWE1 p.R4187C results in increased mutation frequency and impaired oxidative stress response. These alterations may be particularly relevant in cortical progenitor zones and contribute to the XLID onset.

## Results

### Altered HUWE1 protein levels are accompanied with increased mutation frequency in XLID individual cells

**Figure 1:**
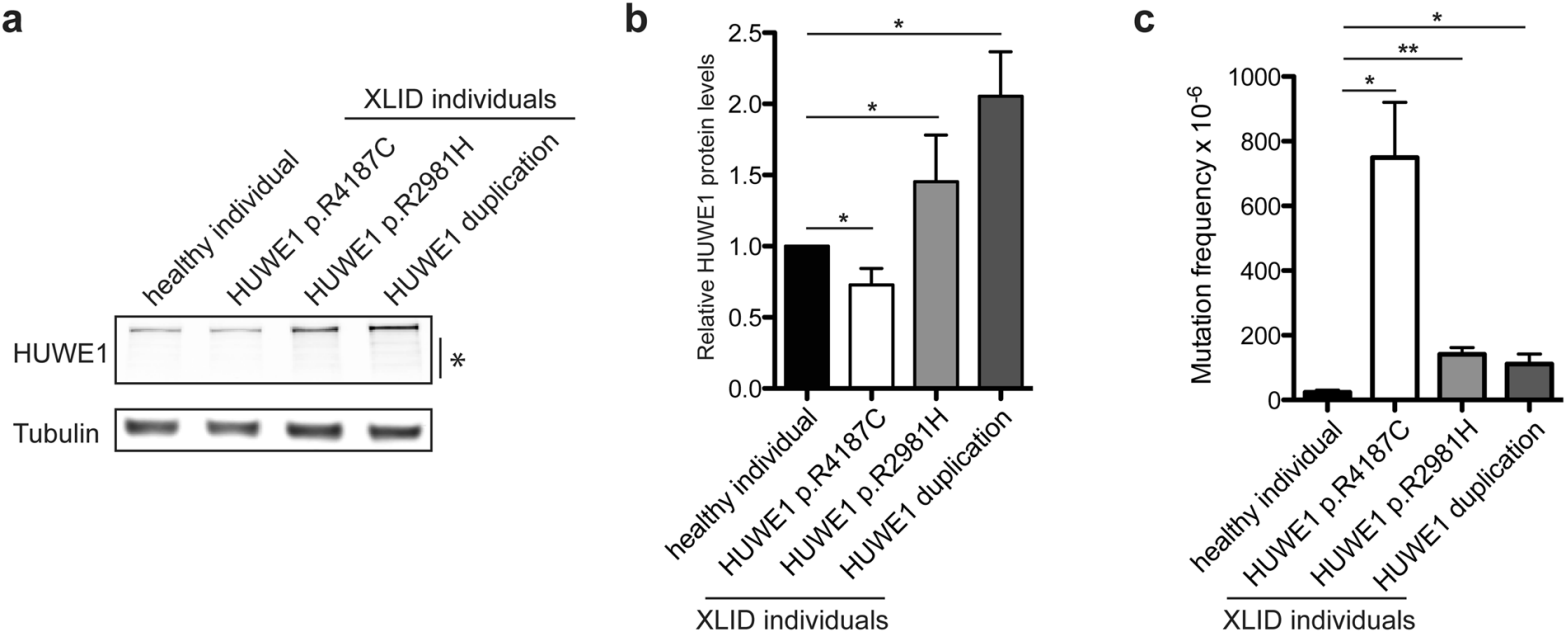
HUWE1 protein levels are altered and mutation frequency increased in HUWE1-promoted XLID cells. (**a**) Immunoblot analysis of HUWE1 protein levels in healthy and the three indicated HUWE1-promoted XLID individual cell lines harboring duplicated HUWE1, HUWE1-R4187C or HUWE1-R2981H. Asterisk indicates unspecific bands. (**b**) Quantification of (**a**) (n = 3). Error bars indicate mean ± SD. The difference in the HUWE1 expression between healthy individual and XLID cells was determined by unpaired t-test, two-tailed p-value; *p < 0.05 and **p < 0.01 – HUWE1 p.R4187C, t(4) = 4.08 p = 0.0151; HUWE1 p.R2981H, t(4) = 3.05 p = 0.0381; HUWE1-duplication t(4) = 4.08 p = 0.0151. (**c**) Endogenous mutation frequency in healthy and three XLID individuals assessed by the PigA-mutation frequency assay (n = 3). Error bars indicate mean ± SEM. The significance of mutation frequency change between genotypes was determined by unpaired t-test, two-tailed p-value; *p < 0.05 and **p < 0.01 – HUWE1 p.R4187C, t(4) = 4.24 p = 0.0133; HUWE1 p.R2981H, t(4) = 5.32 p = 0.006; HUWE1-duplication t(4) = 2.81 p = 0.0486.

To addressed how XLID-specific *HUWE1* mutations and duplication affect the protein levels immunoblot analysis using lymphoblastoid cell lines (LCLs) derived from a healthy individual and three different XLID donors, harboring duplicated *HUWE1*, HUWE1 p.R4187C, or HUWE1 p.R2981H, was performed. While HUWE1 levels were significantly lower in XLID cells with HUWE1 p.R4187C, they were higher in the cells with HUWE1 p.R2981H or duplicated *HUWE1*, when compared to the healthy individual cells (Fig. 1a,b). This indicated that HUWE1 protein levels are altered in XLID individual cells. Since alterations in the HUWE1 levels result in increased genome instability^14^, and accumulation of genomic mutations has been associated with the onset of neurodevelopmental disorders, we next tested if the observed alterations in the HUWE1 levels affect the mutation frequency in XLID cells by using the *PigA*-mutation frequency assay^26^. A strong increase in the mutation rate was observed in all three XLID cell lines (Fig. 1c). Interestingly, the highest increase in the mutation frequency was detected in the XLID cells with the HUWE1 p.R4187C (Fig. 1c), which is expressed at a lower level than HUWE1 wild-type (WT) protein (Fig. 1a,b). Taken together, our findings indicate that the XLID associated genetic alterations affect HUWE1 expression and result in increased genome instability.

### Protein levels of HUWE1 substrate DNA polymerase λ are specifically downregulated in XLID cells

**Figure 2:**
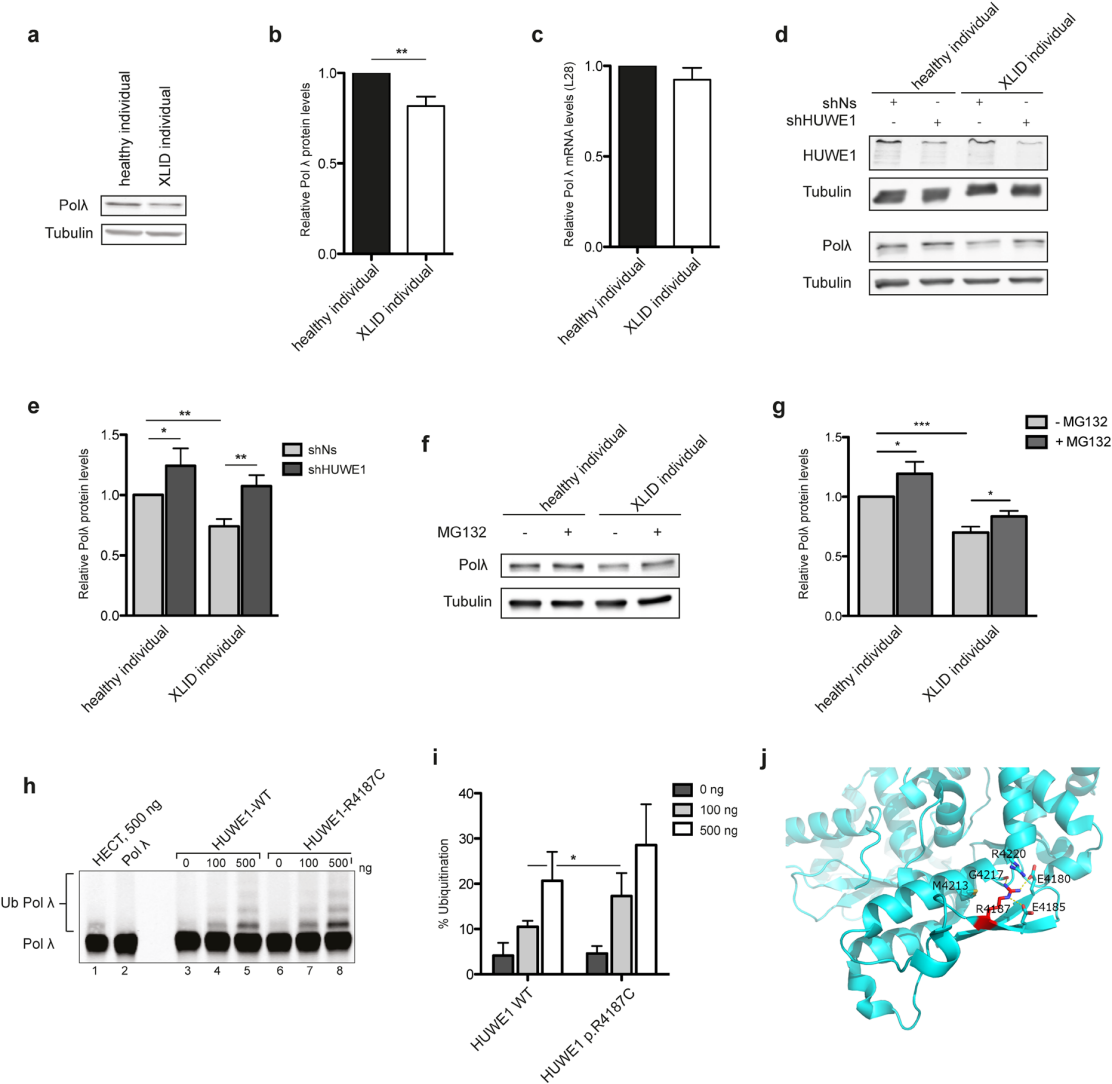
Cellular pool of HUWE1 substrate DNA polymerase λ is specifically reduced in XLID individual cells through HUWE1 p.R4187C hyperubiquitination. (**a**) Immunoblot analysis of the DNA polymerase (Pol) λ levels in healthy and XLID individual (HUWE1-R4187C) cells. (**b**) Quantification of (**a**) (n = 3). Error bars indicate mean ± SD. The significance of protein level changes was determined by unpaired t-test, two-tailed p-value; **p < 0.01 – Polλ t(4) = 6.06 p = 0.0037. (**c**) mRNA levels of Polλ in healthy and XLID individual cells addressed by RT-qPCR (n = 3). Error bars indicate mean ± SD; n.s ≥ 0.05, t(4) = 1.99 p = 0.1169. (**d**) Immunoblot analysis of HUWE1 and Polλ protein levels in healthy and XLID individual cells expressing scrambled (shNs) or HUWE1-targeting (shHUWE1) shRNA. (**e**) Quantification of (**d**) (n = 3). Error bars indicate mean ± SD. The significance of changes in the Polλ protein was determined by unpaired t-test, two-tailed p-value; *p < 0.05 and **p < 0.01 – shNs (healthy individual) vs shHUWE1 (healthy individual) t(4) = 2.95 p = 0.042; shNs (healthy individual) vs shNs (XLID individual) t(4) = 7.4 p = 0.0018; shNs (healthy individual) vs shHUWE1 (XLID individual) t(4) = 5.28 p = 0.0061. (**f**) Immunoblot analysis of Polλ protein levels in healthy and XLID individual cells after 24-hour treatment with 0.625 µM proteasome inhibitor MG132 or dimethyl sulfoxide (DMSO) as a control. (**g**) Quantification of (**f**) (n = 3). Polλ levels were calculated relative to Tubulin. Error bars indicate mean ± SD. The significance of changes in the Polλ protein was determined by unpaired t-test, one-tailed p-value; *p < 0.05, ***p < 0.001–0 µM (healthy individual) vs 0.625 µM (healthy individual) t(4) = 3.24 p = 0.0158; 0 µM (XLID individual) vs 0.625 µM (XLID individual) t(4) = 3.37 p = 0.0141; 0 µM (healthy individual) vs 0 µM (XLID individual) t(4) = 10.51 p = 0.0002. (**h**) *In vitro* ubiquitination of purified recombinant Polλ with the wild type (WT) HUWE1 or mutant ∆2906 HUWE1 p.R4187C. Ub Polλ designates both mono- as well as polyubiquitinated forms. (**i**) Quantification of (**h**) (n = 3). Error bars indicate mean ± SD, unpaired t-test, two-tailed p-value; *p < 0.05; t(4) = 2.22 p = 0.0454. (**j**) Structural analyses of the R4187 residue, based on the recently determined crystal structure of a HUWE1 C-terminus that includes the catalytic domain (PDB ID: 5lp8)^27^. The figure was made using the program PyMol [The PyMol Molecular Graphics System, Version 1.7.2.3, Schrödinger, LLC.].

To determine if increased mutation rate is caused by dysregulation of HUWE1 substrates, which play a role in genome maintenance, an immunoblot screen was performed in healthy individual and XLID cells. From three tested XLID LCLs, HUWE1 p.R4187C cells with highest mutation frequency (Fig. 1c) were chosen for the subsequent analysis (hereafter referred to as XLID individual cells). A significant change in the protein levels of two HUWE1 substrates was observed; Polλ levels were downregulated, while Cdc6 was upregulated in XLID individual cells (Figs 2a,b and S1). The protein levels of other analyzed HUWE1 substrates involved in genome maintenance: MUTYH (MutY homologue), p53, TopBP1 (DNA topoisomerase 2-binding protein (1), BRCA1 (BRCA1, DNA repair associated), HDAC2 (histone deacetylase (2) and c-Myc (v-myc avian myelocytomatosis viral oncogene homolog) did not significantly differ between healthy and XLID individual cells (Fig. S2).

To test if the observed changes in Polλ and Cdc6 occur on the posttranslational level and potentially directly correlate with the HUWE1 p.R4187C, mRNA levels were analyzed next. While no difference in the Polλ mRNA amounts was observed (Fig. 2c), Cdc6 mRNA levels were significantly upregulated in XLID cells when compared to healthy individual (Fig. S2). This suggests that Polλ dysregulation occurs directly on the posttranslational level, and is potentially caused by mutated HUWE1 p.R4187C E3 ubiquitin ligase. In contrast to Polλ, dysregulation of Cdc6 is an indirect result of changes at the level of transcription.

### HUWE1 p.R4187C, mutated in XLID, hyperubiquitinates Polλ and predisposes it for proteasomal degradation

To test if Polλ dysregulation is caused directly by mutated HUWE1 p.R4187C, healthy and XLID individual cells with stably knocked down HUWE1 were generated. Interestingly, upon the HUWE1 knockdown in XLID individual cells Polλ levels were rescued back to the ones observed in the healthy individual cells (Fig. 2d,e), suggesting that Polλ levels are directly modulated by HUWE1 p.R4187C. Similarly, and in line with previous findings^13^, knockdown of HUWE1 in the healthy individual cells led to Polλ accumulation (Fig. 2d,e). To address if the HUWE1-caused changes in Polλ levels were mediated via subsequent proteasomal degradation, healthy and XLID individual cells were treated with a proteasomal inhibitor MG132. The MG132 treatment resulted in stabilization and significant increase of the Polλ protein levels both in healthy and XLID individual cells (Fig. 2f,g). This indicated that HUWE1 p.R4187C downregulates Polλ levels in XLID individual cells by predisposing it for proteasomal degradation. Next, to determine if Polλ downregulation is a result of altered HUWE1 activity caused by p.R4187C mutation, which resides within catalytic HECT domain, *in vitro* ubiquitination assays were performed. The activity of ∆2906 HUWE1 WT and p.R4187C was compared in reactions with purified human Polλ, E1 and E2 enzymes. Interestingly, HUWE1 p.R4187C more efficiently ubiquitinated Polλ than HUWE1 WT (Fig. 2h,i), thus suggesting that p.R4187C mutation renders HUWE1 hyperactive. These findings are supported by the observed reduction in HUWE1 and Polλ levels in XLID individual cells (Figs 1a and 2a), since increased ubiquitination of the two proteins results in promoted proteasomal degradation^9–11,13^. To further explore the importance of the amino acid R4187, structural analysis of recently published C-terminal HUWE1 structure^27^ was performed (Fig. 2j). The HUWE1 C-terminal HECT domain adopts a bilobal structure, with the C-terminal lobe containing the catalytic cysteine that participates in the transfer of ubiquitin to substrate, and the N-terminal lobe representing the E2 binding domain. In this uncomplexed structure of the C-terminal HECT domain, the affected R4187 residue is located in the beta hairpin within the E2-binding region in the N-terminal lobe. As shown in Fig. 2j, the R4187 stretches towards the helical core of the protein and is engaged in several hydrogen bonds. A R4187C mutation will likely affect the interaction with the E2 enzymes and consequently influence the ubiquitin transfer. In summary, these findings indicate that the HUWE1 p.R4187C is hyperactive towards Polλ, resulting in XLID-specific Polλ downregulation.

### Oxidative stress response is impaired in XLID individual cells

**Figure 3:**
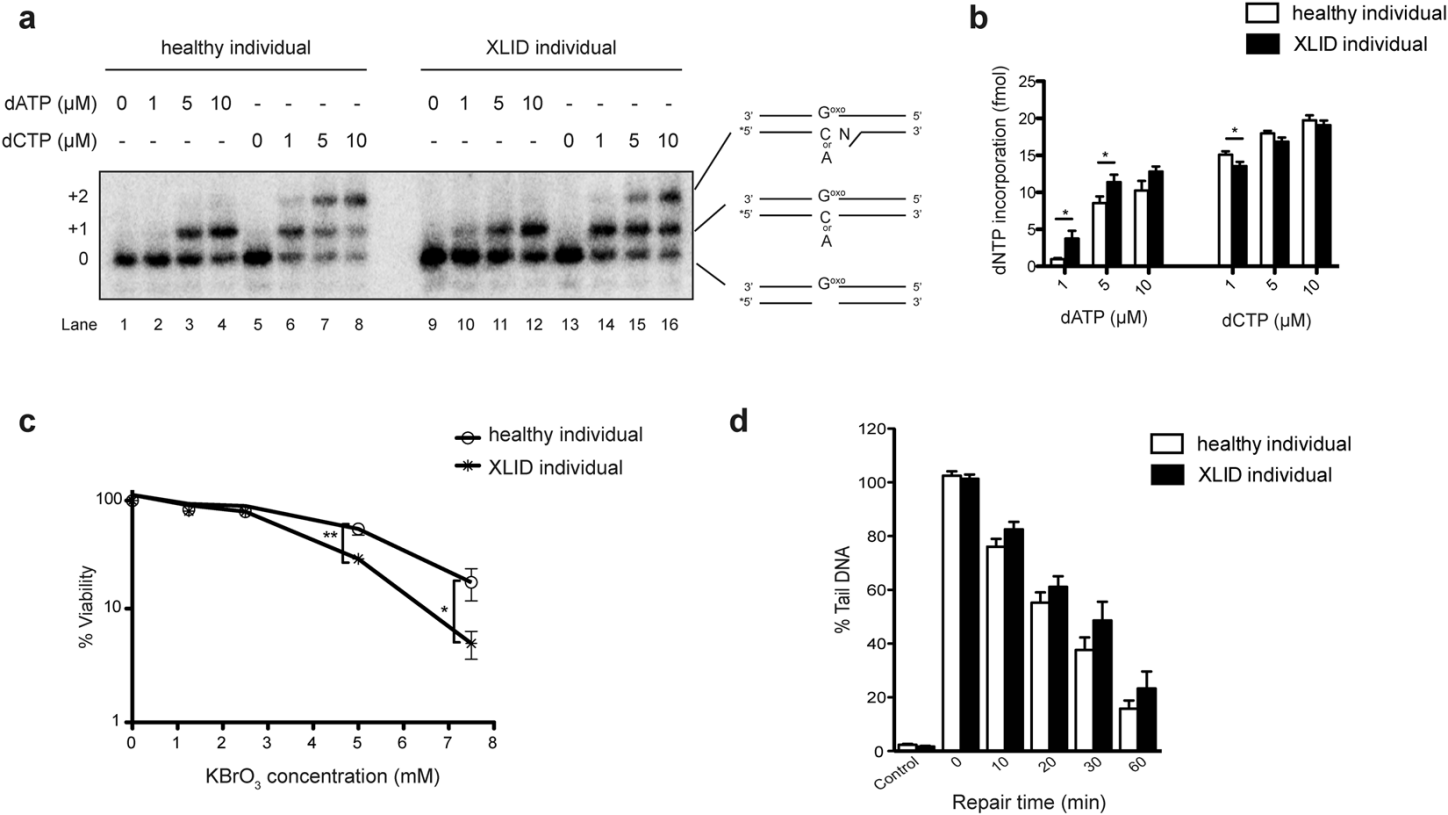
Oxidative stress response is impaired in HUWE1 p.R4187C XLID individual cells. (**a**) Efficiency and accuracy of dATP versus dCTP incorporation opposite frequent oxidative DNA lesion 7,8-dihydro-8-oxo-guanine (G^oxo^), using increasing amounts of healthy and XLID individual (HUWE1-R4187C) whole cell extracts (WCEs) in an *in vitro* DNA polymerase assay. (**b**) Quantification of (**a**) (n = 5). Error bars indicate mean ± SEM. The significance of changes between healthy and XLID individual WCEs was determined by unpaired t-test, one-tailed p-value; *p < 0.05–1 µM dATP t(7) = 2.29 p = 0.0277; 5 µM dATP t(8) = 2.11 p = 0.034; 1 µM dCTP t(7) = 2.4 p = 0.0238. (**c**) Cell viability of healthy and XLID individual (HUWE1-R4187C) cells after 24 hours of continuous treatment with increasing concentrations of potassium bromate (KBrO_3_) (n = 3). The significance of changes in the survival upon the treatment was determined by unpaired t-test, two-tailed p-value; n.s ≥ 0.05, *p < 0.05 and **p < 0.01. (**d**) Single-cell gel electrophoresis (Comet) analysis of DNA repair kinetics upon H_2_O_2_-treatment in healthy and XLID individual cells, represented as the relative percentage of DNA in the tail. Mean of 3 independent experiments presented. In each experiment at least 100 cells were analyzed per each time point and each cell type. Error bars indicate mean ± SEM.

Our findings indicate that XLID individual cells have increased mutation frequency and reduced Polλ levels (Figs 1 and 2). Interestingly, Polλ has been suggested to play an important role in mutagenesis by counteracting C:G→A:T transversion mutations that arise from the frequent oxidative lesion 7,8-dihydro-8-oxo-guanine (8oxoG)^13,28–31^. Lack of Polλ was shown to cause increased misincorporation of dATP and reduced incorporation of dCTP opposite 8oxoG. This leads to accumulation of A:8oxoG mispairs that subsequently give rise to C:G→A:T mutations in the next round of replication, or undergo futile cycles of DNA repair with toxic single strand breaks as byproducts^32^. To determine if reduced Polλ levels in XLID individual cells affect 8oxoG bypass fidelity an *in vitro* DNA polymerase assay was performed (Fig. 3a,b). A Polλ-specific DNA substrate that contains a single nucleotide gap facing an 8oxoG lesion in the complementary strand was used. As indicated in Fig. 3a the incorporation of dCTP decreased, while misincorporation of dATP increased in the XLID whole cell extracts (WCEs), when compared to extracts from healthy individual cells. The observed differences were most significant at the lower dNTP concentrations: 1 μM and 5 μM dATP and 1 μM dCTP (Fig. 3b), which is consistent with the role of Polλ in gap filling at lower dNTP concentrations^33^. Taken together these data indicate that the presence of oxidative base damage 8oxoG in the genome of HUWE1-promoted XLID cells has high mutagenic potential that is accompanied with reduced Polλ levels. In addition to increased inaccurate 8oxoG bypass, reduction in Polλ levels was shown to cause hypersensitivity to oxidative stress^29^. To test if XLID individual cells with reduced Polλ levels have altered sensitivity to oxidative stress, potassium bromide (KBrO_3_) treatment was performed and viability determined. The XLID individual cells were hypersensitive to oxidative stress induced by KBrO_3_, when compared to the cells from healthy individual (Fig. 3c). The hypersensitivity of XLID cells was oxidative stress-specific, as no difference in the sensitivity to alkylation treatment was detected between healthy and XLID individual cells (Fig. S3). Subsequent Comet analysis showed increased accumulation of toxic single and double strand breaks as well as labile abasic (AP) sites at all time points after exposure to the oxidizing agent hydrogen peroxide (H_2_O_2_) in the XLID cells as compared to healthy individual cells (Fig. 3d). In summary, these findings indicate that exposure to oxidative stress results in hypersensitivity of HUWE1 p.R4187C XLID individual cells, potentially caused by accumulation of DNA breaks and labile AP sites.

### Inhibition of USP7 efficiently reverses the oxidative stress hypersensitivity of XLID cells

**Figure 4:**
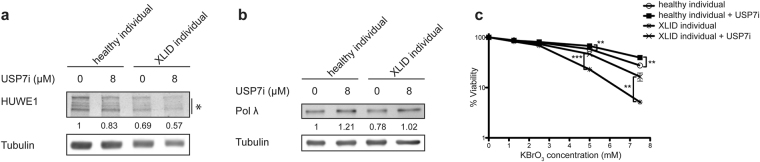
USP7 inhibition reverses the XLID-specific hypersensitivity to oxidative stress. (**a**) Immunoblot analysis of HUWE1 and Polλ protein levels in healthy and XLID individual (HUWE1-R4187C) cells treated for 24 hours with 8 µM P5091, the USP7 inhibitor (USP7i), or DMSO as a control. Asterisk indicates unspecific bands. (**b**) Quantification of (**a**) (n = 3). (**c**) Cell viability of healthy and XLID individual cells pretreated for 24 hours with 8 µM USP7i or DMSO as a control, followed by a continuous 24 hour treatment with increasing concentrations of potassium bromate (KBrO_3_) (n = 3). Error bars indicate mean ± SD. The significance was determined by unpaired t-test, two-tailed p-value; **p < 0.01 and ***p < 0.001–5 mM KBrO3 (healthy individual vs healthy individual + USP7i) t(4) = 5.96 p = 0.004, (XLID individual vs XLID individual + USP7i) t(4) = 13.59 p = 0.0002; 7.5 mM KBrO3 (healthy individual vs healthy individual + USP7i) t(4) = 8.23 p = 0.0012, (XLID individual vs XLID individual + USP7i) t(4) = 6.42 p = 0.003.

HUWE1 levels are directly regulated by USP7S that efficiently de-ubiquitinates HUWE1 and prevents its subsequent degradation^10^. We hypothesized that by inhibiting USP7S it would be possible to promote HUWE1 p.R4187C degradation and consequently stabilize Polλ, as well as rescue the defects observed in XLID cells, like hypersensitivity to oxidative stress. To test this hypothesis, healthy and XLID individual cells were treated with P5091, a USP7 inhibitor (USP7i) currently in preclinical trials^34^. Treatment with USP7i resulted in decreased HUWE1 protein levels (Fig. 4a) as well as stabilization and increase in Polλ protein levels (Fig. 4b) both in healthy and XLID individual cells. Importantly, besides the impact on the protein level, USP7i treatment resulted in reversal of the XLID hypersensitivity to the oxidizing agent KBrO_3_ to the level comparable to the healthy individual cells (Fig. 4c). These findings support the idea that the hypersensitivity to oxidative stress observed in XLID individual cells is directly cased by HUWE1 p.R4187C, potentially through Polλ.

### Human HUWE1 localizes in the regions resembling cortical progenitor zones

**Figure 5:**
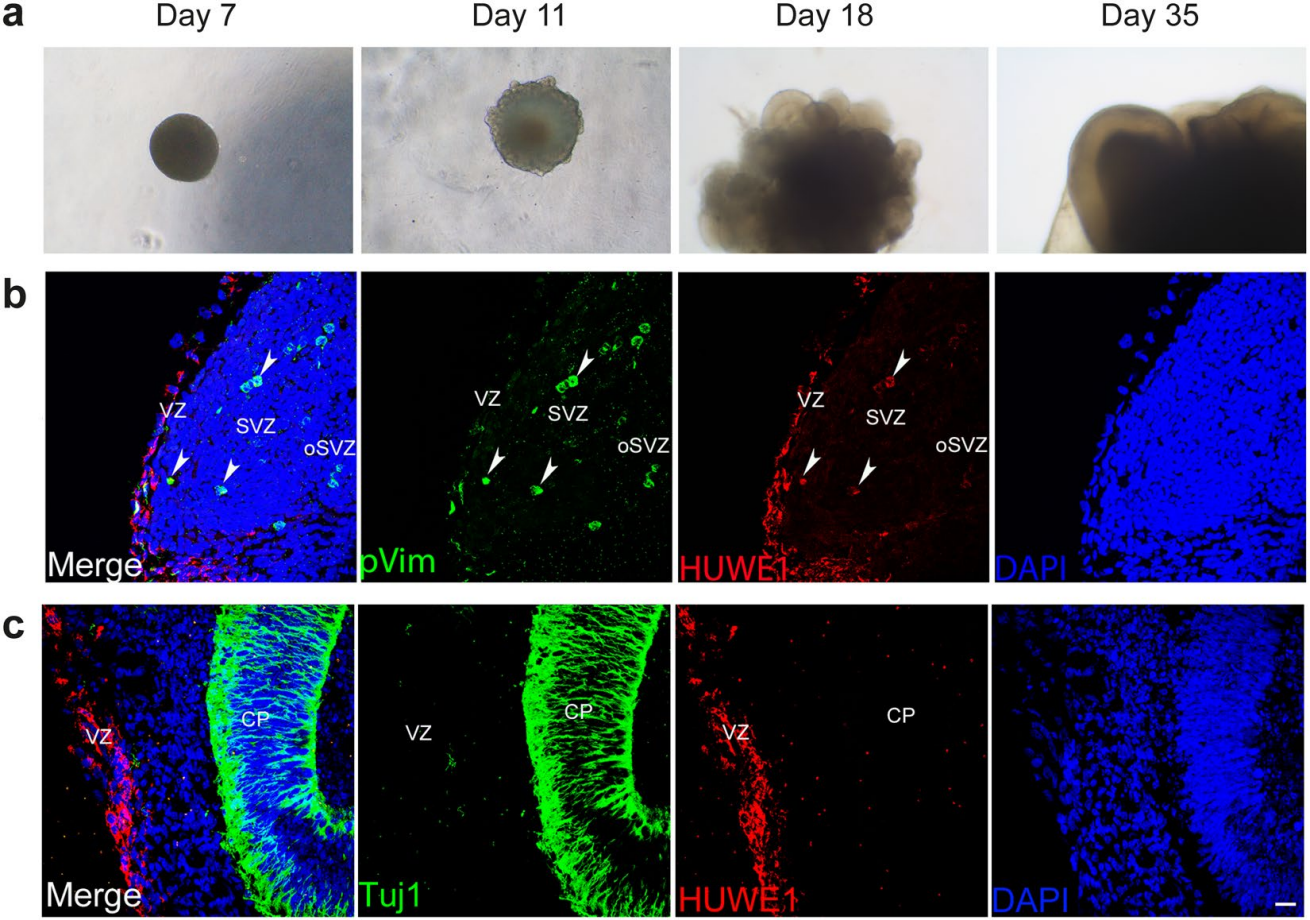
Human HUWE1 localizes in ventricular and subventricular zones. (**a**) Representative bright-field images of cerebral organoids at 7, 11, 18 and 35 days of differentiation. (**b**,**c**) Confocal microscope images of 35-days old cerebral organoids stained with DAPI, and antibodies against HUWE1, Phospho-vimentin (pVim) a marker of radial glia cells (**b**), and Tuj1 a marker of differentiated neurons (**c**). HUWE1 co-localizes with pVim (arrowheads) in ventricular (VZ), subventricular (SVZ) and outer SVZ (oSVZ), but not with Tuj1 in cortical plate (CP). Scale bar, 10 µm.

Several studies using mouse models demonstrated an essential role of HUWE1 in murine neurodevelopment^18,20^. Currently it is however not clear where in the human brain HUWE1 localizes and to which extent it contributes to human neurodevelopment. To explore HUWE1 distribution in human cortical structures and to obtain insight in which regions HUWE1 misregulation could particularly contribute to the development of XLID, we developed cerebral organoids by differentiating healthy individual hiPSCs (Fig. 5a). Immunofluorsecence analysis of 35-days old cerebral organoids revealed that human HUWE1 is mainly expressed in cortical progenitor regions, resembling ventricular and sub-ventricular zones (VZ and SVZ, respectively) (Fig. 5b). Further HUWE1 co-localized with phospho-Vimentin (pVim), a marker of mitotic radial glia, NSCs. Interestingly, in 35-days old organoids HUWE1 was not detectable in the Tuj1 positive neurons (Fig. 5c). These results suggest that human HUWE1 localizes in the regions resembling cortical progenitor zones where, through its essential role in neurodevelopment, could contribute to the onset of XLID.

## Discussion

Mutations in more than 100 XLID-causing genes have been identified during four decades of research. Besides high genetic variability, a high clinical heterogeneity has been reported in individuals with different XLID syndromes^2^. The high degree of heterogeneity and limited number of individuals with identical mutations in XLID causative genes present a particular challenge for identification of molecular processes altered in XLID. In this study we identified cellular pathways impaired in XLID cells and define regions resembling human cortex that are potentially most affected by mutated HUWE1. Our findings show that XLID-causing *HUWE1* mutations lead to alterations in HUWE1 protein levels and result in increased mutation frequency in particular in XLID cells with reduced HUWE1 levels (Fig. 1). This is in line with a very recent observation that loss of HUWE1 causes genome instability^14^. The increase in the mutation frequency, observed in XLID cells, similar to previous studies^35,36^, suggests that the rate of *de novo* mutations may be higher in individuals with XLID than in healthy individuals. Further by analyzing HUWE1 substrates involved in genome maintenance we observe a specific downregulation of DNA repair enzyme Polλ on posttranslational level by hyperactive HUWE1 p.R4187C (Fig. 2). The observation that p.R4187C mutation results in altered HUWE1 activity (Fig. 2) is in line with the very recent findings obtained in *C*. *elegans*, which showed that the same mutation in EEL-1 (HUWE1 homologue) does not rescue GABE-ergic presynaptic transmission to the same extent as does the WT EEL-1^22^. In addition to Polλ, HUWE1 p.R4187C could catalyze hyper-selfubiquitination that causes reduced HUWE1 protein levels, as observed in Fig. 1.

Polλ was previously shown to play an important role in counteracting mutagenesis caused by the oxidative DNA base damage 8oxoG and reduction in its protein levels is known to cause hypersensitivity to oxidative stress^13,28–31^. The hypersensitivity is thought to result from increased dATP misincorporation opposite 8oxoG that can lead to futile 8oxoG repair cycles, a byproduct of which are toxic single strand breaks. In line with this, we show that HUWE1-promoted XLID cells with reduced Polλ levels incorporate more dATP opposite 8oxoG and display hypersensitivity to oxidative stress (Figs 1 and 3). Contribution of altered DNA repair to onset of IDs has been suggested through identification of causative mutations in several DNA repair genes^16,17^. Besides increase in the mutation frequency and altered DNA repair, the hypersensitivity to oxidative stress might be particularly relevant for the onset of HUWE1-promoted XLID, as enhanced oxidative stress and insufficient antioxidant defense were suggested to play important pathophysiological role in XLID^24,25^.

Given the high oxygen metabolism in the brain, the observed hypersensitivity to oxidative stress and the increased mutation frequency potentially contribute to an altered neuronal development in HUWE1-promoted XLID. This could be particularly relevant in cortical progenitor regions, where we show, by using cerebral organoids, that human HUWE1 localizes in pVim positive mitotic radial glia cells (Fig. 5). The observed localization further supports a conserved role of HUWE1 in neurodevelopment, as previously suggested by *HUWE1*-knockout mice studies^18,20,21^. Loss of HUWE1 in the mouse brain caused an enlarged NSC compartment followed by disorganization of the cortical layers, revealing a crucial function of HUWE1 in triggering the maturation of neuronal progenitors residing in the cortical germinal layers. Further HUWE1 inactivation in mouse radial glia and cerebellar granule neuron precursors indicated an important function of HUWE1 in synchronization of neuronal and glial differentiation^37^. Besides developing cerebral organoids from hiPSCs harboring HUWE1 WT we also attempted to develop cerebral organoids from HUWE1 p.R4187C hiPSCs. However, selection of HUWE1 p.R4187C containing hiPSCs proved to be inefficient (data not shown). Since HUWE1 is known to control NSC activity^20^, it is possible that XLID-specific HUWE1 mutations would affect stem cell properties, which would hamper the selection of hiPSCs with mutated HUWE1. Follow up study addressing this is ongoing.

Overall, in this study we for the first time provide evidence that XLID-causing HUWE1 mutation leads to changed catalytic activity and results in alterations of essential cellular processes, such as maintenance of genome stability and response to oxidative stress, which could be relevant in cortical progenitor zones of human brain, as suggested by HUWE1 localization. By elucidating processes altered in HUWE1 p.R4187C XLID we provide first insights into molecular basis of this complex neurodevelopmental condition.

## Methods

### Cell culture

XLID individual LCLs were published previously^5^, while the healthy individual LCL (AG09387) was obtained from the Coriell Cell Repository (Coriell Institute for Medical Research, USA). LCLs were maintained in RPMI 1640 (Life Technologies) with 15% FCS (Life Technologies), 1% penicillin/streptomycin (Life Technologies), under 5% CO_2_ atmosphere at 37 °C. Human induced pluripotent stem cells (hiPSCs) ATCC-DYS0100 were obtained from ATCC (Manassas, USA) and were grown in mTeSR1 medium (STEMCELL Technologies) on CellMatrix Basement Membrane Gel coated dishes (ATCC), under 5% CO_2_ atmosphere at 37 °C.

### Whole cell extracts

Lymphoblastoid cells growing in suspension were collected and washed twice with ice cold PBS, followed by flash freezing of the cell pellet in liquid N_2_. Subsequently, the thawed cells were resuspended in one packed cell volume (PCV) of lysis buffer I (10 mM Tris-HCl pH 7.8, 200 mM KCl, 1 mM NEM, 0.1 mM MG-132, 1 μM PMSF, 2 μM leupeptin, 3 μM bestatin, 1.5 μM pepstatin) and two PCV of lysis buffer II (10 mM Tris-HCl pH 7.8, 600 mM KCl, 2 mM EDTA, 40% glycerol, 0.2% NP-40, 1 mM NEM, 0.1 mM MG-132, 1 μM PMSF, 2 μM leupeptin, 3 μM bestatin, 1.5 μM pepstatin). The samples were further rotated for 30 minutes at 4 °C, sonicated and centrifuged. The supernatants representing WCEs were either flash frozen in liquid N_2_ and stored at −80 °C or used for analysis directly.

### Immunoblot analysis

Whole cell extracts were either separated on a 7% Tris-Acetate polyacrylamide (PA) gel (HUWE1, BRCA1) or on a 10% Tris-Glycine PA gel and transferred to an Immobilon-FL membrane (Millipore) for subsequent immunoblotting. Primary antibodies were detected using infrared (IR) Dye-conjugated secondary antibodies (Rockland). The signal was visualized using direct IR fluorescence via the Odyssey Scanner, LI-COR Biosciences. Primary antibodies: BRCA1 (sc-6954, Santa Cruz), Cdc6 (ab155759, Abcam), c-Myc (sc-789, Santa Cruz), HDAC2 (sc-9959, Santa Cruz), HUWE1 (A300-486A, Bethyl Laboratories), p53 (sc-6243, Santa Cruz), Polλ (A301-640A-1, Bethyl Laboratories), TopBP1 (NB100-217, Novus Biologicals), Tubulin (T9026, Sigma-Aldrich). All original images of immunoblots are depicted in Supplementary Fig. S1.

### RNA isolation and quantitative PCR

LCLs were resuspended in TRI reagent® (Molecular Research Center, Inc.), mixed with 1/5 volume of chloroform, incubated for 3 min at room temperature and spun down at 12,000 × g. The transparent phase was separated and the RNA was precipitated with isopropanol and washed once with 75% ethanol. The RNA pellet was further air-dried and dissolved in H_2_O-DEPEC. Subsequently, cDNA was generated with the SuperScript® III Reverse Transcriptase (Technologies) according to the manufacturers protocol. Quantitative PCR (qPCR) was performed with a Rotor Gene RG3000A, Corbett Research using gene specific primer and the SensiMix™ SYBR® Hi-ROX Kit (Bioline). qPCR was performed with following oligonucleotides (Microsynth): Polλ(fwd)5′-CCTGTGCCCTGCTCTACTTC-3′ and Polλ(rev)5′-CTCAGCAGGTTCTCGGTAGG-3′; Cdc6(fwd)5′-GGGAATCAGAGGCTCAGAAG-3′ and Cdc6(rev)5′-CACTGGATGTTTGCAGGAGA-3′; L28(fwd)5′-GCAATTCCTTCCGCTACAAC-3′ and L28(rev)5′-TGTTCTTGCGGATCATGTGT-3′. Results were normalized against L28 expression.

### *PigA*-locus mutation frequency assay

The mutation frequency assay was performed as described in^26^. LCLs growing in suspension were collected and washed twice with ice cold staining buffer (1x HBSS, 0.1% (w/v) NaN_3_, 1% (w/v) BSA) and brought to a concentration of 10^7^ cells/ml in staining buffer. Cells were incubated with the fluorescent antibodies against CD48 (PE mouse anti-human, 552855, BD Pharmingen), CD59 (PE mouse anti-human, 555764, BD Pharmingen) and HLA-DR (FITC mouse anti-human, 555560, BD Pharmingen) and incubated for 20 min on ice in the dark. Finally, the cells were washed twice with staining buffer and brought to a concentration of 2.5 × 10^6^ cells/ml in staining buffer. Immediately before analysis 25 µl propidium iodide (PI) solution (50 µg/ml PI, 0.1% (w/v) sodium citrate) per ml were added to exclude dead cells. The flow cytometry was performed on a LSRII Fortessa, BD Biosciences. Per condition 1 × 10^6^ live cells were analyzed, cells negative for CD48/59 but positive for HLA-DR were considered mutants.

### *In vitro* ubiquitination

*In vitro* ubiquitination assay were performed similar as previously described by Markkanen *et al*.^38^. Briefly, ubiquitin, E1 ubiquitin-activating enzyme and the three E2 ubiquitin-conjugating enzymes UbcH5b/5c/7 (U-Boston Biochem) were pre-mixed with ATP (Sigma-Aldrich), recombinant Polλ and 10x ubiquitin buffer (250 M Tris-HCl pH 8, 50 mM MgCl_2_, 2 mM CaCl_2_, 10 mM DTT, 100 µM MG-132). To this pre-mix, increasing amounts of GST-tagged recombinant ΔN2906 HUWE1 (WT-AG or R4187C) were added. The *in vitro* ubiquitination was performed at 30 °C for 60 min, stopped with SDS-PAGE loading dye and separated on a NuPAGE® Novex® 4–12% Bis-Tris Protein Gels (Life Technologies) and immunoblotted as described above. Primary Polλ antibody was generated in our laboratory.

### Structural analysis

Structural analysis was done using the program PyMol [The PyMol Molecular Graphics System, Version 1.7.2.3, Schrödinger, LLC.]

### 8oxoG specificity assay

The 8oxoG specificity assay was performed as published previously^39^. Briefly, a 39-mer upstream primer (γP^32^-labeled) and a 60-mer downstream oligonucleotide were annealed to a 100mer template containing an 8oxoG lesion (X) at position 61, thereby generating a single-nucleotide gap opposite the 8oxoG (39-mer: 5′-TACAACCAAGAGCATACGACGGCCAGTGCCGAATTCACA-3′; 60-mer: 5′-GGTGTTGTGTGTTGGTTGTGGTGGTGTTGTGTGGTTGTTGGTGTTGTGTGTGTTGGTGTG-3′; 100-mer: 5′-CACACCAACACACACAACACCAACAACCACACAACACCACCACAACCAA CACACAACACCXTGTGAATTCGGCACTGGCCGTCGTATGCTCTTGGTTGTA-3′). The reaction mixture (10 µl) was composed of 1 mM MgCl_2_, 1x polymerase buffer (100 mM Tris-HCl pH 7.5, 1 mg/ml BSA, 0.1 mM DTT), 2 µg trap-DNA (5′-AAAAAAAAAAAAAAAAAAAAAAAAAAAAAAAAAAA-3′), 10 µg aphidicolin, 2 nM substrate-DNA, 1 µl dNTPs and 5 µg WCE. Aphidicolin was added into reaction to inhibit replicative Pols and specifically test the activity of translesion Pols. Samples were incubated for 30 min at 37 °C. The reactions were stopped with 10 µl stop buffer (95% formamide, 10 mM EDTA, xylene cyanol, and bromophenol blue), heated for 5 min at 95 °C, separated on a 12.5% PA-urea gel (3.5 h at 90 W) and visualized on a Typhoon phosphoimager.

### Cell viability assay

LCLs were seeded in a 96-well plate (100,000 cells/well) in complete medium and treated with increasing concentration of KBrO_3_ or methyl methanesulfonate (MMS), as indicated in the figures. Each condition was run in a technical quadruplicate. After 24 h of incubation, the cell viability was addressed applying PrestoBlue® (Life Technologies) according the manufacturers protocol and the absorbance was analyzed with the infinite® M200, Tecan plate reader.

### Alkaline comet assay

Initially, LCLs were treated with 75 µM hydrogen peroxide (H_2_O_2_) for 15 min in RPMI 1640 (Life Technologies) at 37 °C. After the treatment, the cells were washed once with cold PBS, resuspended in complete medium and plated for recovery. At each indicated time point (0–60 min) the cells were collected, washed with cold PBS and stored on ice at a concentration of 200,000 cells/ml PBS. After all samples were collected, the cells were mixed at 37 °C 1/10 with 1% low melting point agarose (Lonza). For each condition, 20 µl of the agarose/cell suspension were spread per well on a CometAssay® HT slide (Trevigen). Samples were prepared in technical duplicates. The agarose droplets were left to solidify for 10 min at 4 °C before placed into the pre-cooled lysis solution (2.5 M NaCl, 100 mM EDTA pH 10, 10 mM tris base, 1% sarcosyl, 1% triton X-100) for 60 min at 4 °C in the dark. Subsequently, the slides were incubated twice for 10 min in pre-cooled alkaline solution (200 mM NaOH, 1 mM EDTA pH 8) for DNA unwinding and breakage of the alkali-labile sites, before subjected to electrophoresis in fresh alkaline solution for 20 min at 0.3 A. Finally, the slides were washed twice in ddH_2_O, once in 70% ethanol and then left to air-dry. To visualize the DNA, the slides were stained with SYBR® Green I (Sigma-Aldrich) (in TE pH 8) for 15 min at 4 °C and washed twice for 10 min with ddH_2_O. The dried slides were analyzed using the Comet Assay IV (Perceptive Instruments) system.

### shRNA knockdown

Wild-type (WT) LCLs were lentivirally transduced with plasmids carrying the non-specific control shRNA (pGIPZ-Ns, Open Biosystems) or the Mule-targeting shRNA (GIPZ Human HUWE1 shRNA, Clone-ID V3LHS_353153, TCCTCTGTACCAACAACCT, Open Biosystems). Stable clones were subsequently selected with puromycin (1 µg/ml), while the selection-efficiency was monitored by the percentage of GFP-positive cells.

### USP7 inhibitor treatment

Cells were treated with 8 µM of the USP7 inhibitor p5091 (Selleckchem) for 24 h before they were further used for a KBrO_3_-viability assay or cell pellets were collected for WCE preparation and subsequent immunoblotting.

### Cerebral organoids

hiPSCs have been differentiated to cerebellar organoids following protocol published by Lancaster and Knoblich^40^. Briefly, feeder-independent hiPSCs were dissociated and allowed to reaggregate to form embryonic bodies, which were then subjected to neural induction in minimal medium allowing neuroectoderm formation. The neuroectodermal tissue was next transferred to Matrigel and upon outgrowth of neuroepithelaial buds transferred to spinning bioreactor. 35-days after the initiation of differentiation cerebral organoids were collected and prepared for cryosectioning.

### Immunofluorescence analysis

The 18-µm thick cryosections were immunostained following previously described procedure^41^. Briefly, upon drying sections were incubated in the blocking solution (PBS + 0.25% Triton + 10% goat serum) for 1 h at RT. Next primary antibody diluted in PBS + 0.25% Triton + 10% goat serum was added and sections incubated over night at 4 °C. After four washes with washing buffer (PBS + 0.25% Triton), secondary antibody diluted in PBS + 0.25% Triton was added and slides incubated for 2 h at room temperature. Slides were next washed four times with washing buffer, followed by two short washes with ddH2O. Primary antibodies: phosphorylated Vimentin (Ser55) (D076-3S, MBL International), TUJ1 (MMS-435P, Covance) and HUWE1 (A300-486A, Bethyl Laboratories). Secondary antibodies: Goat anti-Rabbit IgG (H + L) Secondary Antibody, Alexa Fluor® 568 conjugate (A-11011, Thermo Fischer) and Goat anti-Mouse IgG (H + L) Secondary Antibody, Alexa Fluor® 488 conjugate (A-11029, Thermo Fischer). Microscopy was carried out using a Leica SP8 confocal microscope equipped with ×40 oil immersion lens, using Huygens software.

### Statistical analysis

Differences in the endogenous mutation frequency, protein and mRNA levels, dNTP incorporation and the cell viability upon exposure to damaging agents were assessed using unpaired Student t-test in Prism 5.

## Electronic supplementary material

Supplementary Information
