# Publisher Correction: Impaired oxidative stress response characterizes HUWE1-promoted X-linked intellectual disability

**DOI:** 10.1038/s41598-018-24189-2

**Published:** 2018-04-12

**Authors:** Matthias Bosshard, Rossana Aprigliano, Cristina Gattiker, Vuk Palibrk, Enni Markkanen, Paul Hoff Backe, Stefania Pellegrino, F. Lucy Raymond, Guy Froyen, Matthias Altmeyer, Magnar Bjørås, Grigory L. Dianov, Barbara van Loon

**Affiliations:** 1 Department of Molecular Mechanisms of Disease, University of Zurich, Zürich, 8057 Switzerland; 2 Department of Clinical and Molecular Medicine, Norwegian University of Science and Technology (NTNU), Trondheim, 7491 Norway; 3 Institute of Clinical Medicine, Faculty of Medicine, University of Oslo, Oslo, 0318 Norway; 4 Department of Medical Genetics, Cambridge Institute for Medical Research, Cambridge, CB2 0XY United Kingdom; 5 Human Genome Laboratory, Department of Human Genetics, Leuven, 3000KU Belgium; 6 Department of Pathology and Medical Genetics, St. Olavs Hospital, Trondheim University Hospital, Trondheim, 7491 Norway; 7 CRUK/MRC Institute for Radiation Oncology, Department of Oncology, University of Oxford, Oxford, OX3 7DQ United Kingdom; 8 Institute of Cytology and Genetics, Siberian Branch of the Russian Academy of Sciences, Novosibirsk, 630090 Russia

Correction to: *Scientific Reports* 10.1038/s41598-017-15380-y, published online 08 November 2017

In the original version of this Article, the author Paul Hoff Backe was incorrectly indexed. This error has now been corrected.

