# Supplementary material for: Impaired oxidative stress response characterizes HUWE1-promoted X-linked intellectual disability
Source: Sci Rep. 2017 Nov 8;7:15050. doi: 10.1038/s41598-017-15380-y (PMC5678168; doi:10.1038/s41598-017-15380-y)
Supplement: Supplementary file 1 — Supplementary Information [file 41598_2017_15380_MOESM1_ESM.pdf]

**SUPPLEMENTARY INFORMATION**

# TITLE: Impaired oxidative stress response characterizes HUWE1-promoted X-linked intellectual disability

**Authors:** Matthias Bosshard[1], Rossana Aprigliano[1,2], Cristina Gattiker[1], Vuk Palibrk[2], Enni Markkanen[1], Paul Hoff Backe[3], Stefania Pellegrino[1], F. Lucy Raymond[4], Guy Froyen[5], Matthias Altmeyer[1], Magnar Bjørås[2,3,6], Grigory L. Dianov[7,8], Barbara van Loon[1,2,6,*]

**Affiliations:**

[1]Department of Molecular Mechanisms of Disease, University of Zurich, Zürich 8057, Switzerland

[2]Department of Clinical and Molecular Medicine, Norwegian University of Science and Technology (NTNU), Trondheim 7491, Norway

[3]Institute of Clinical Medicine, Faculty of Medicine, University of Oslo, Oslo 0318, Norway

[4]Department of Medical Genetics, Cambridge Institute for Medical Research, Cambridge CB2 0XY, United Kingdom

[5]Human Genome Laboratory, Department of Human Genetics, Leuven 3000KU, Belgium

[6]Department of Pathology and Medical Genetics, St. Olavs Hospital, Trondheim University Hospital, Trondheim 7491, Norway

[7]CRUK/MRC Institute for Radiation Oncology, Department of Oncology, University of Oxford, Oxford OX3 7DQ, United Kingdom

[8]Institute of Cytology and Genetics, Siberian Branch of the Russian Academy of Sciences, Novosibirsk 630090, Russia

*corresponding author, e-mail: barbara.v.loon@ntnu.no.

## SUPPLEMENTARY FIGURE LEGENDS

**Fig. S1. Original immunoblots related to Fig. 1a, 2a, 2d, 2f, 2g, 4a and 4b.**

**Fig. S2. Expression of HUWE1 substrates in XLID individual cells.**

(*a*) Immunoblot analysis of HUWE1 substrates: MUTYH (MutY homologue), p53, TopBP1 (DNA topoisomerase 2-binding protein 1), BRCA1 (BRCA1, DNA repair associated), HDAC2 (histone deacetylase 2) and c-Myc (v-myc avian myelocytomatosis viral oncogene homolog) in healthy and XLID individual (HUWE1 p.R4187C) cells. (b) Quantification of Cdc6 protein levels from independent extracts by immunoblot analysis, as the one represented in (a) (n=3). The significance of protein level changes was determined by unpaired t-test. Error bars indicate mean ± SD; **$p<0.01$, $t(4)=6.86$ $p=0.0024$. (c) Cdc6 mRNA levels in healthy and XLID individual cells addressed by RT-qPCR (n=3). Error bars indicate mean ± SD; **$p<0.01$, $t(4)=4.69$ $p=0.0094$.

**Fig. S3. XLID and healthy individual cells are equally sensitive to alkyltion treatment.** Cell viability of healthy and XLID individual (HUWE1 p.R4187C) cells after 24 hours of continuous treatment with increasing concentrations of methyl methanesulfonate (MMS) (n=3).

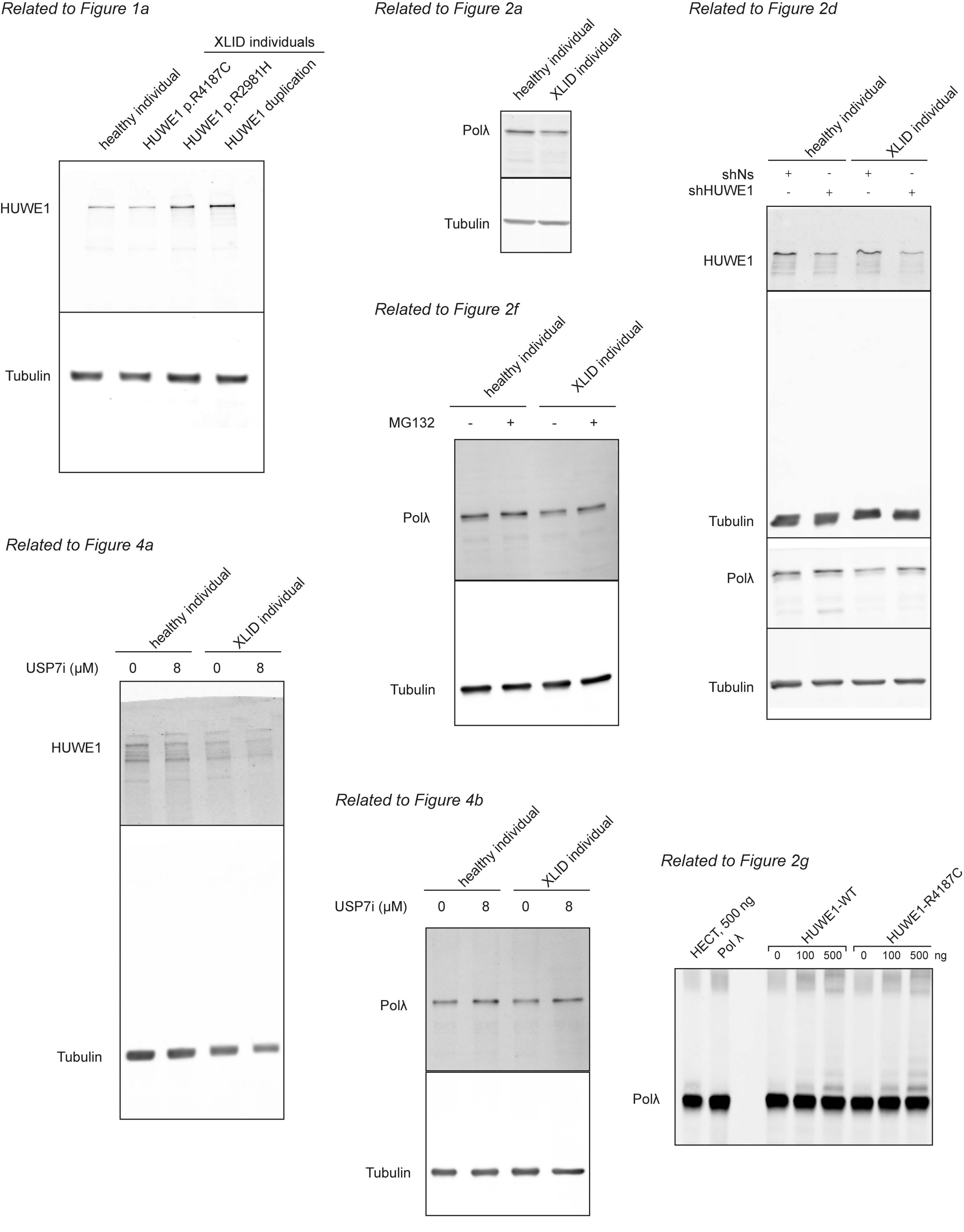
Related to Figure 1a
XLID individuals
healthy individual
HUWE1 p.R4187C
HUWE1 p.R2981H
HUWE1 duplication
HUWE1
Tubulin
Related to Figure 2a
healthy individual
XLID individual
Polλ
Tubulin
Related to Figure 2d
healthy individual
XLID individual
shNs + - + -
shHUWE1 - + - +
HUWE1
Tubulin
Polλ
Tubulin
Related to Figure 2f
healthy individual
XLID individual
MG132 - + - +
Polλ
Tubulin
Related to Figure 4a
healthy individual
XLID individual
USP7i (µM) 0 8 0 8
HUWE1
Tubulin
Related to Figure 4b
healthy individual
XLID individual
USP7i (µM) 0 8 0 8
Polλ
Tubulin
Related to Figure 2g
HECT, 500 ng
Pol λ
HUWE1-WT
HUWE1-R4187C
0 100 500 0 100 500 ng
Polλ

**Figure S1**

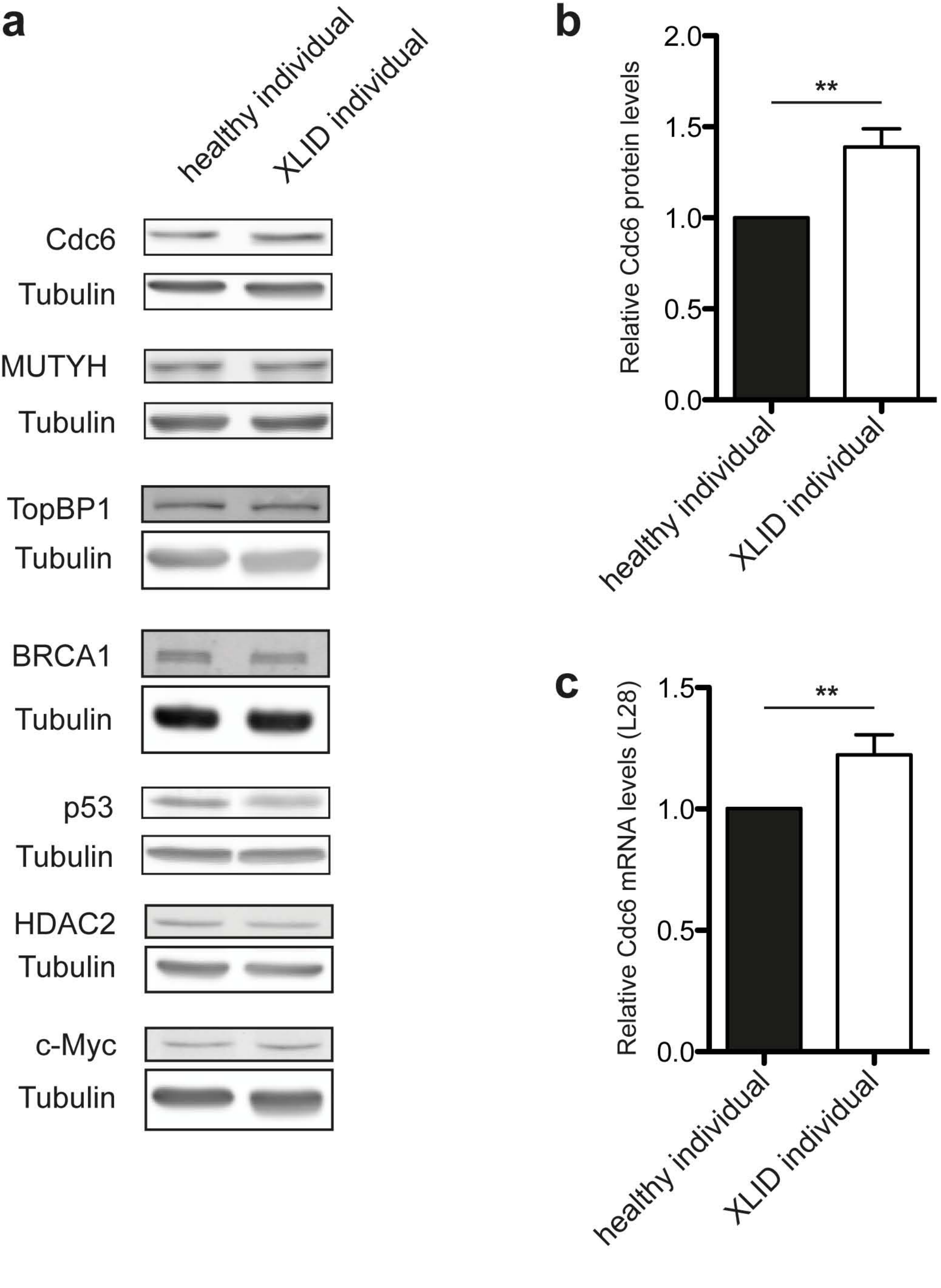
a
healthy individual
XLID individual
Cdc6
Tubulin
MUTYH
Tubulin
TopBP1
Tubulin
BRCA1
Tubulin
p53
Tubulin
HDAC2
Tubulin
c-Myc
Tubulin
b
Relative Cdc6 protein levels
2.0
1.5
1.0
0.5
0.0
**
healthy individual
XLID individual
c
Relative Cdc6 mRNA levels (L28)
1.5
1.0
0.5
0.0
**
healthy individual
XLID individual

**Figure S2**

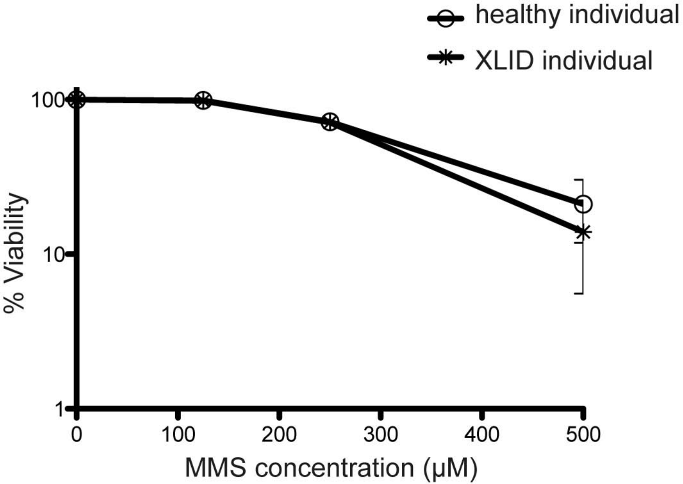
healthy individual
XLID individual
100
10
1
% Viability
0
100
200
300
400
500
MMS concentration (µM)

# Figure S3
